# Cannons and sparrows II: the enhanced Bernoulli exact method for determining statistical significance and effect size in the meta-analysis of *k* 2 × 2 tables

**DOI:** 10.1186/s12982-021-00101-8

**Published:** 2021-08-03

**Authors:** Lawrence M. Paul

**Affiliations:** Bell Laboratories, Somerset, New Jersey 08873 United States

**Keywords:** Meta-analysis, Categorical analysis, Mantel–Haenszel, DerSimonian, Exact solution, Inverse variance, Convolution, Heterogeneity, Rare events

## Abstract

**Background:**

The use of meta-analysis to aggregate the results of multiple studies has increased dramatically over the last 40 years. For homogeneous meta-analysis, the Mantel–Haenszel technique has typically been utilized. In such meta-analyses, the effect size across the contributing studies of the meta-analysis differs only by statistical error. If homogeneity cannot be assumed or established, the most popular technique developed to date is the inverse-variance DerSimonian and Laird (DL) technique (DerSimonian and Laird, in Control Clin Trials 7(3):177–88, 1986). However, both of these techniques are based on large sample, asymptotic assumptions. At best, they are approximations especially when the number of cases observed in any cell of the corresponding contingency tables is small.

**Results:**

This research develops an exact, non-parametric test for evaluating statistical significance and a related method for estimating effect size in the meta-analysis of *k* 2 × 2 tables for any level of heterogeneity as an alternative to the asymptotic techniques. Monte Carlo simulations show that even for large values of heterogeneity, the Enhanced Bernoulli Technique (EBT) is far superior at maintaining the pre-specified level of Type I Error than the DL technique. A fully tested implementation in the R statistical language is freely available from the author. In addition, a second related exact test for estimating the Effect Size was developed and is also freely available.

**Conclusions:**

This research has developed two exact tests for the meta-analysis of dichotomous, categorical data. The EBT technique was strongly superior to the DL technique in maintaining a pre-specified level of Type I Error even at extremely high levels of heterogeneity. As shown, the DL technique demonstrated many large violations of this level. Given the various biases towards finding statistical significance prevalent in epidemiology today, a strong focus on maintaining a pre-specified level of Type I Error would seem critical. In addition, a related exact method for estimating the Effect Size was developed.

“Little experience is sufficient to show that the traditional machinery of statistical processes is wholly unsuited to the needs of practical research. Not only does it take a cannon to shoot a sparrow, but it misses the sparrow. The elaborate mechanism built on the theory of infinitely large samples is not accurate enough for simple laboratory data.” (R. A. Fisher, 1925)

## Background

**Fig. 1:**
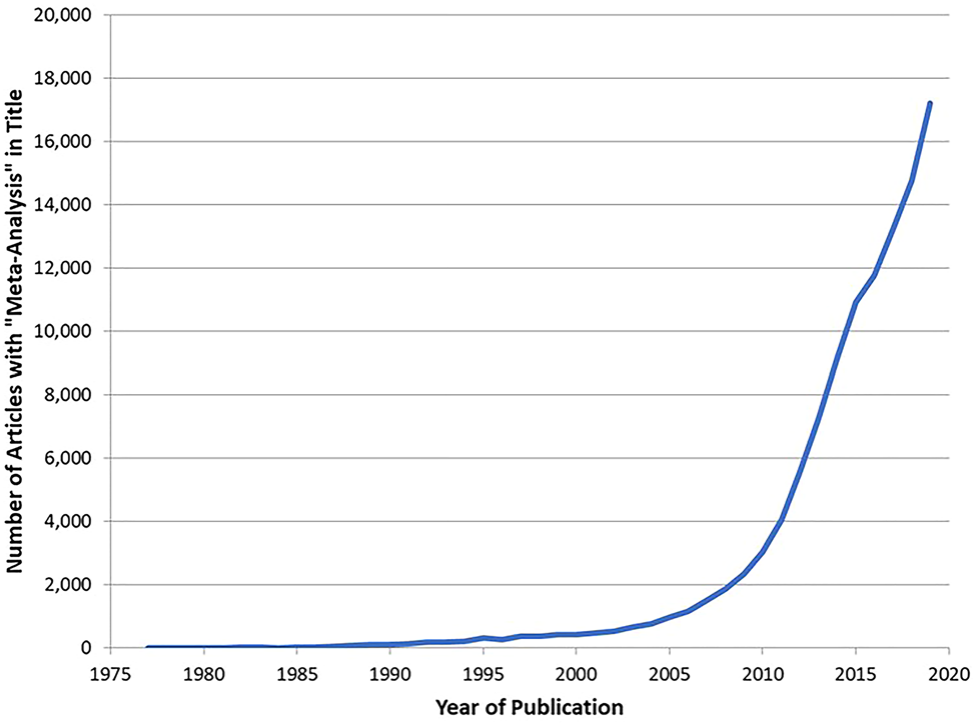
Number of articles containing “meta-analysis” in the title by year of publication

The use of meta-analysis in epidemiological research has been increasing at a very rapid rate. A review of the National Library of Medicine’s online database (“Pub Med”) shows that in 1977 there was only a single research article with the term “meta-analysis” in its title. This number had increased to 138 in 1991, 966 in 2005 and to 17,205 in 2019 (see Fig. 1).

Part of this growth may be due to the widespread availability of powerful personal computer software making meta-analysis techniques more feasible to implement. More importantly, the need to draw meaningful conclusions from an aggregation of small studies may help explain this exponential growth.

The use of meta-analytic techniques is controversial when the contributing studies are not randomized control trials (RCT). Many researchers feel that it is highly misleading to attempt to combine a series of disparate studies [1] while others maintain that, with proper safeguards, meta-analysis allows an extremely useful pooling of smaller studies [2, 3]. A discussion of the appropriateness of meta-analysis is beyond the scope of this paper. Rather, the focus here will be on minimizing unnecessary error in testing the overall statistical significance of a meta-analysis and in estimating the Effect Size.

### Overview of 2 × 2 × k categorical meta-analysis

The “2 × 2 × k” categorical meta-analysis paradigm is probably the most frequently encountered situation in meta-analysis. It consists of a series of *k* contributing studies each described by a 2 × 2 contingency table. Every cell of each 2 × 2 table contains the number of occurrences of an event (e.g., disease case) for the particular combination of row and column variables. For the sake of illustration, we can associate the two columns of each table with Disease Manifestation vs. No Disease Manifestation and the two rows with Exposure vs. No Exposure. Table 1 represents the results of one of these *k* studies.

**Table 1:** Typical contributing study (one of *k*) in a dichotomous meta-analysis

Exposure	Disease status		Total
	Disease manifestation	No disease manifestation	
Exposure	4	96	100
No exposure	2	98	100
Total	6	194	200

In most meta-analyses, there are typically two distinct components: (1) A statistical test of the overall difference between the Exposure and No Exposure groups across the *k* contributing studies; and (2) A method to pool the observed differences between groups across the *k* studies in order to estimate the true difference (the Effect Size).

Surprisingly, in recent years, many epidemiologists employing meta-analytic techniques have greatly deemphasized the first component. Borenstein et al*.* [2] conclude:

“…However, meta-analysis also allows us to move beyond the question of statistical significance, and address questions that are more interesting and also more relevant.” (pp. 11–12).

Similarly, Higgins et al*.* [3] rather dismissively state:

“…If review authors decide to present a p value with the results of a meta-analysis, they should report a precise p value, together with the 95% confidence interval” (pp. 371–372).

A method is developed that maintains the Type I error (“false alarm rate”) at the desired level, but which has good power to detect true differences across a large range of event probability, number of contributing studies, sample size and level of heterogeneity. An argument can be made that maintaining the Type I error at a pre-specified level is more important than the power (1—Type II error rate) to detect true differences between conditions. The framers of modern statistical testing called such errors “Errors of the First Kind” and placed a special emphasis on them. Neyman and Pearson in 1933 stated:

“A new basis has been introduced for choosing among criteria available for testing any given statistical hypothesis, H_*0*_, with regard to an alternative H_*t*_*.* If ϴ_*1*_ and ϴ_*2*_ are two such possible criteria and if in using them there is the same chance, ε, of rejecting H_*0*_ when it is in fact true, we should choose that one of the two which assures the minimum chance of accepting H_*0*_ when the true hypothesis is H_*t*_.” [4] (p. 336).

Thus, while Neyman and Pearson supported the effort to choose criteria that yield the greatest power to detect true differences, this effort is secondary to maintaining a pre-specified level of Type I error. A second exact method is developed to estimate the effect size of any statistically significant finding.

### “Rare” events and meta-analysis

The probability of occurrence of a disease is often categorized as “rare” although no specific definition exists. As an example, Higgins et al*.* state that “There is no single risk at which events are classified as ‘rare’”, but gives as examples 1 in a 100 or 1 in a 1000 (see [5], p. 520). An obvious related issue is observing zero cases in one or more cells of a contingency table. Table 2 shows the expected cell sizes from various realistic combinations of disease probability and contributing study sample size.

**Table 2:** Expected number of disease cases as a function of disease probability and individual study sample size (each arm)

Disease/condition	Approximate disease prob	Individual study sample size (each arm)		
		100	500	1000
Myocardial infarction incidence rate (Age ≥ 60 years)	0.011 [25]	1.1	5.5	11
Parkinson’s disease incidence rate (60–69 age group)	0.00058 [26]	0.06	0.29	0.58
Alzheimer’s disease incidence rate (60–74 age group)	0.002 [27]	0.2	1	2
Lung cancer incidence rate (White Males)	0.00051 [28]	0.05	0.26	0.51

Table 2 supports the notion that “rare” events are a focus of many epidemiological studies.

For homogeneous meta-analysis (i.e., where the effect across studies may be assumed to be the same within statistical variation), the two techniques typically used for categorical data are the Mantel–Haenszel and Peto techniques. Both of these techniques rely on the Mantel–Haenszel Chi Square to test for the overall statistical significance. For heterogeneous meta-analyses, the asymptotic DerSimonian–Laird (DL) inverse variance technique is typically used [6].

The problem in applying large sample asymptotic techniques to meta-analyses involving small numbers of cases will be illustrated in the older and much more developed domain of homogeneous meta-analyses. Mantel developed what is probably the most widely used technique for homogeneous meta-analyses [7]. In applying his technique, he showed that a minimum of approximately five cases was required in each of the four cells of each of the 2 × 2 tables for each of the *k* studies comprising the meta-analysis [8]. This is the same heuristic requirement typically used without any particular justification for the simple chi-square test. Mantel and Fleiss reviewed the options when a reasonable number of cases was not present in all cells:

“The investigators could have obtained data from very many more tables to make things more asymptotic for use of M–H [note: this is the Mantel–Haenszel technique], or they could readily have applied a more exact procedure for the data at hand” (p. 134).

R. A. Fisher made essentially the same plea in 1925 in the preface to the first edition of his well-known *Statistical Methods for Research Workers* [9]:

“Little experience is sufficient to show that the traditional machinery of statistical processes is wholly unsuited to the needs of practical research. Not only does it take a cannon to shoot a sparrow, but it misses the sparrow. The elaborate mechanism built on the theory of infinitely large samples is not accurate enough for simple laboratory data. Only by systematically tackling small sample problems on their merits does it seem possible to apply accurate tests to practical data.”

Both criticisms suggest the use of exact methods to handle the sparseness of the underlying contingency tables at least for the disease examples contained in Table 2. All but two of the combinations of individual study sample size and disease probability shown in Table 2 would yield fewer than five cases per cell leading to violations of the minimum cell size in the Mantel–Haenszel (MH) Chi Square test, and thus the test would be potentially flawed. In addition, these two cases were for sample size equal to 500 and 1000 which probably don’t represent many realistic studies. While this limitation of the MH Chi Square test was known to Mantel and others (e.g., [8]), it seems to generally have been forgotten for meta-analysis of 2 × 2 × k categorical data. The continued use of an asymptotic test in situations not suited for its use is unacceptable given the computer power that is now available to all researchers.

### Heterogeneity vs. homogeneity in meta-analyses

The term “heterogeneity” refers to the fact that studies done at different times and by different researchers might be expected to yield different results. The expectation is that a variable of interest may be dependent, at least in part, on one or more other variables. The meta-analysis researcher, J. P. T. Higgins stated “As Heterogeneity is to be expected in a meta-analysis: it would be surprising if multiple studies, performed by different teams in different places with different methods, all ended up estimating the same underlying parameter.” ([10], p. 158). While researchers may agree that heterogeneity is to be expected, there is very little agreement on how to quantify this variability. The obvious candidate is τ^2^, the estimated variability between studies. However, τ^2^ is not invariant across study designs and its interpretation may not be intuitive. Alternatives include $I^{2}$, the ratio of the inter-study variability to the total variability and the *Q* statistic, which is mathematically related to $I^{2}$ (see, e.g., [11]).

In this paper, heterogeneity will be mathematically manipulated through τ^2^ using the logit distribution as developed by Bhaumik et al. [12]. Namely:

1 $$x_{Ci} \sim B\left( {p_{Ci,} n_{Ci} } \right), x_{Ei} \sim B\left( {p_{Ei,} n_{Ei} } \right),$$

2 $$ \log it\left( {p_{Ci} } \right) = \mu + \varepsilon_{1i} , \log it\left( {p_{Ei} } \right) = \mu + \theta + \varepsilon_{1i} + \varepsilon_{2i} $$

3 $$\varepsilon_{1i} \sim N\left( {0,\gamma^{2} } \right)$$

4 $$\varepsilon_{2i} \sim N\left( {0,\tau^{2} } \right)$$

where B is the Binomial Distribution; N is the Normal Distribution; x_*Ci*_, x_*Ei*_ are the observed number of cases in the control and exposure groups respectively of the ith study; p_*Ci*_, p_*Ei*_ are the event probabilities in the control and exposure groups respectively of the ith study; n_*Ci*_, n_*Ei*_ are the sample sizes in the two groups of the ith study; µ corresponds to the background event (disease) probability in the exposure and control groups; $\theta$ is the logarithm of the overall ratio of the event probability in the exposure group to the event probability in the control group; $\gamma^{2}$ is the variance corresponding to the uncertainty of the observed disease probability in both the exposure and control groups of the *k* contributing studies; $\tau^{2}$ is a variance corresponding to the heterogeneity which exists only in the exposure group across the *k* contributing studies; $\varepsilon_{1i}$ is a Normal distribution deviation in background event (disease) probability for both the exposure and control groups of the *ith* contributing study and $\varepsilon_{2i}$ is a Normal distribution deviation in background disease probability due to heterogeneity only in the exposure group for the *ith* contributing study.

### The basic principles of the Dersimonian–Laird (DL) method

As stated above, this research develops an exact method for conducting meta-analyses in *k* 2 × 2 tables with heterogeneity and contrasts it with the most popular approach which was developed by DerSimonian and Laird (DL) [6].

For each contributing study, the DL technique calculates the logarithm of the sample odds ratio and a corresponding estimate of the variance of this measure based on the asymptotic distribution of these logarithms. Adjustments are made for entries in the individual 2 × 2 tables that contain a zero-cell count. Equations 5–8 below capture the core DL approach. In Eq. 5, an estimate of the interstudy variability, $\tau^{2}$, is first derived from Cochran’s Q statistic and the weights assigned to each of the *k* contributing studies, $\omega_{i}$. Each weight is equal to the inverse of the variance of the estimated fixed effect log odds ratio, $\hat{\theta }_{i } ,$ for that contributing study.

5 $$\hat{\tau }^{2} = \frac{{Q - \left( {k - 1} \right)}}{{\sum \omega_{i} - \left( {\frac{{\sum \omega_{i}^{2} }}{{\sum \omega_{i} }}} \right)}}$$

As shown in Eq. 6, a new set of weights, $\omega_{i}^{^{\prime}}$, are then calculated based on the estimated value of $\hat{\tau }^{2}$ from Eq. 5 and the standard errors of the contributing studies.

6 $$\omega_{i}^{^{\prime}} = \frac{1}{{SE\left( {\hat{\theta }_{i} } \right)^{2} + \tau^{2} }}$$

These new weights are then used to calculate estimates of both the overall log odds ratio, $\theta_{DL}$ and its standard error as shown in Eqs. 7 and 8.

7 $$\hat{\theta }_{DL } = \frac{{\sum \omega_{i}^{^{\prime}} \hat{\theta }_{i } }}{{\sum \omega_{i}^{^{\prime}} }}$$

8 $$SE\left( {\hat{\theta }_{DL} } \right) = \frac{1}{{\sqrt {\sum \omega_{i}^{^{\prime}} } }}$$

A test of statistical significance is then based on a large sample normal distribution. The DL technique requires asymptotic assumptions regarding both the Q statistic used to estimate the interstudy variability, $\tau^{2} ,$ and the normal distribution required to test for statistical significance. A more subtle issue is the possibility of distorting correlations between the individual estimates of the effect size for each contributing study, $\theta_{i}$, and the individual weights used for each of these contributing effect sizes.

## Results

### A non-parametric exact test of overall statistical significance for dichotomous categorical meta-analysis

Jakob Bernoulli’s notion of what is now called a Bernoulli Trial offers the basis for a non-parametric approach to aggregating multiple epidemiological studies based on dichotomous categorical data. The enhancements to the Bernoulli method developed in this paper offer a practical exact method for assessing the overall statistical significance. A related technique is developed below to estimate the effect size of a dichotomous meta-analysis.

One of the many important contributions of this outstanding seventeenth century mathematician was the idea of the fixed probability of an event over a sequence of independent trials which led to what is now called Bernoulli Trials and to the related Binomial Distribution. In brief, Bernoulli viewed a set of statistical events as a series of independent coin flips with each flip having a probability p of obtaining a head and q = 1 − p of obtaining a tail. This hypothetical coin is often treated as a fair coin where both p and q equal 0.5. The simplest Bernoulli Trials approach encompasses a series of *n* flips and answers questions of the type: what is the probability of observing × heads in n such flips? (See for example Rosner [13]). In epidemiology, one could consider each of the *k* contributing studies of a meta-analysis as a single Bernoulli Trial with p = 0.5. Then the combination of the *k* studies could be analyzed as a binomial distribution. This is the standard Sign Test (see, for example, [14]).

For example, for a meta-analysis of 20 studies, if 15 out of 20 studies had more cases in the exposure group than in the control group, we could ask: What is the probability that 15 or more of the 20 studies could have shown a larger effect in the exposure group strictly by chance alone? If this cumulative probability is less than a pre-specified level of Type I error (e.g., 0.05), one would reject the null hypothesis and conclude there probably exists a statistically reliable relationship between exposure and the end point used.

The principal reason that this approach has seen little use in practical epidemiology is that it suffers from two critical deficits. First, the dichotomous Bernoulli heads vs. tails approach doesn’t deal with the third possibility of a tie. The author of this study believes that no truly useful method to date has been offered to deal with those situations when there are an identical number of events in each of the exposure and the control arms of a study other than to discard the study. Second, a truly exact EBT method requires a complete convolution of the frequency distributions of the contributing studies in order to derive the combined frequency distribution. Even for equal sample size, each of the *k* contributing studies could have a different Bernoulli probability, *p,* requiring a full convolution to determine the null distribution of the total number of times there were more cases in the exposure group relative to the control group across the *k* contributing studies. Before dealing with the ties problem, the determination of the combined distribution will be outlined.

#### Combining the individual studies contributing to the meta-analysis

A critical problem is finding a method for combining the individual study binomial distributions of the *k* contributing studies each with a possibly different *p* value into an overall frequency distribution.

Prior to the widespread availability of computing power, the convolution of a large number of individual binomial distributions was typically handled by approximate methods given the unwieldy nature of the calculations. Even with the advent of available computer power, convolution is still often impractical. As an example, for a meta-analysis involving 24 studies each with a unique binomial distribution, there are over 2 million unique combinations of the studies that need to be considered just to calculate the single discrete probability that exactly 12 of the 24 studies have more cases in the exposure group than in the control group.^1^ However, an exact algorithm was laid out in a readily implementable fashion by Butler and Stephens in a 1993 technical report [15] which can easily be implemented even on a personal computer. The algorithm yields the exact probability distribution of the convolution of individual binomial distributions which in the present application would correspond to the specific studies contributing to a meta-analysis. The method makes use of a recurrence relationship inherent in the binomial distribution which allows the semi-automatic calculation of its probabilities without resort to the simple but overwhelmingly inefficient enumeration of all of the possible combinations of studies. This easily established relationship can be stated as:

$$P\left( {X = 0} \right) = \left( {1 - p} \right)^{n} \;if\; j = 0$$

$$P\left( {X = j} \right) = \left\{ {\frac{{\left( {n - j + 1} \right)}}{j}} \right\} \times \left\{ {\frac{p}{{\left( {1 - p} \right)}}} \right\} \times P\left( {X = j - 1} \right)\;if\,j \ge 1$$

**Fig. 2:**
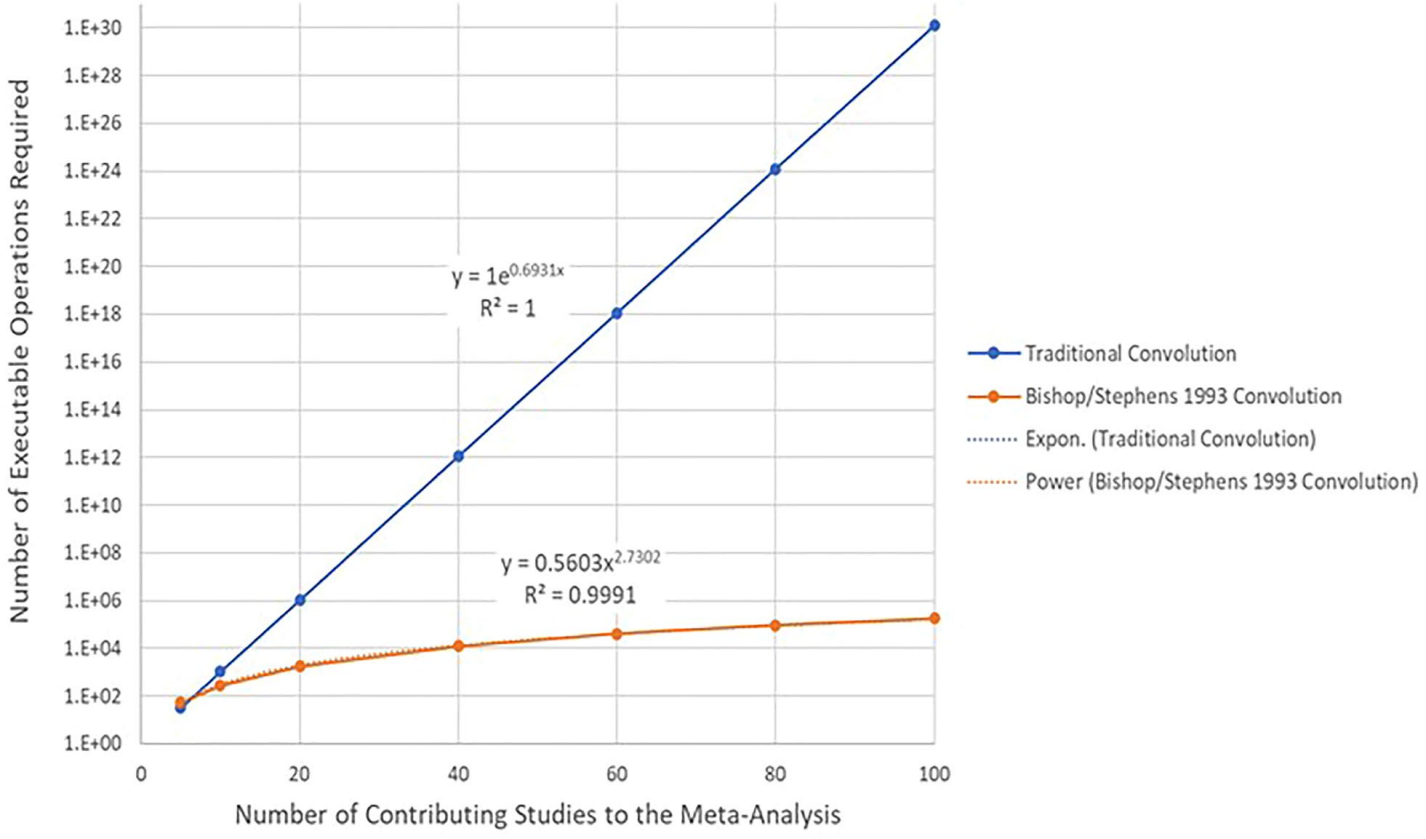
Estimated computer executable steps per Butler and Stephens vs. traditional convolution

Figure 2 compares the estimated number of computer executable steps required in the Butler and Stephens method relative to a traditional convolution.

As can be seen, a traditional convolution is only tractable when the number of contributing studies is less than or equal to approximately 20.

#### The ties problem

The next problem in adapting the standard Bernoulli Trials technique to practical meta-analysis is a procedure to deal with the situation where there are an identical number of cases in both the exposure and control arms of a study contributing to the meta-analysis. In studies with small sample sizes and/or low disease probabilities, the highest probability tie is typically the “0/0” tie in which no cases are observed in either the exposure or the control arms.

A first step in dealing with ties is to more clearly define the criteria for a “success”. The present EBT approach defines a success as there being a *strictly* greater number of cases in the exposure group relative to the control group. Under this definition, the same number of cases in both arms of the study or more cases in the control arm of the study is considered a “failure”. In essence, this is a trinomial situation. There are successes, failures and ties. We are simply combining the failures where there are more cases in the control group relative to the exposure group and tie situations and calling the combination “failures”.

Equation 9 below forms the basis of the EBT method. The Greek capital letter “Π” has been chosen to specify the probabilities of there being more cases in one arm of the study relative to the other to differentiate these parameters from the underlying disease probabilities:

9 $$\Pi_{{E_{i} }} + \Pi_{{C_{i} }} + prob \left( {tie} \right)_{i} = 1$$

Where $\Pi_{{E_{i} }}$ = probability of there being strictly more cases in the exposure group relative to the control group in Study *i; *$\Pi_{{C_{i} }}$ = probability of there being strictly more cases in the control group relative to the exposure group in Study *i; prob(tie)*_*i*_ = probability of finding exactly same number of cases in both groups of Study *i.*

Assuming that $\Pi_{{E_{i} }}$ and $\Pi_{{C_{i} }}$ would be equal under the null hypothesis of no difference between exposure and control groups and rearranging terms, we have:

10 $$2\Pi_{{E_{i} }} + prob\left( {tie} \right)_{i} = 1$$

Solving for $\Pi_{{E_{i} }}$ we have:

11 $$\Pi_{{E_{i} }} = \frac{{1 - prob\left( {tie} \right)_{i } }}{2}$$

Thus, the only requirement for calculating the $\Pi_{{E_{i} }}$ parameter for each contributing study is to first determine the probability of all tie situations for that study.

This is a very straightforward procedure. To determine $prob\left( {tie} \right)_{i}$ for each of the contributing studies, all of the tie situations need to be enumerated and then their probabilities summed together.

As a simple example, assume that Study *i* has 100 participants in each of its exposure and control arms and that the underlying event (disease) probability *p* is 0.01.

The probability that there are no cases among these 100 participants in the exposure arm would then be:

$$Prob\left( {0 cases} \right) = 0.01^{0} \times \left( {1 - 0.01} \right)^{100} = 0.99^{100} = 0.37$$

Similarly, the probability of there being no cases in the control arm would also be 0.37.

Thus, the probability of a “0, 0” tie would be $0.37^{2} = 0.13$ which is surprisingly large.

Table 3 lists the probabilities for the first five tie situations and sums these probabilities to determine $prob\left( {tie} \right)_{i }$.^2^

**Table 3:** Probability of observing exactly the same number of cases in both the exposure and control groups for background event probability equal to 0.01 and sample size equal to 100 as a function of the number of observed cases

Number of cases in each group	Probability
0	0.13
1	0.137
2	0.034
3	0.004
4	0.0002
5	0.00001
Total	0.309

As shown in Table 3, there is over a 30% probability of obtaining a tie for zero cases through five cases in both the exposure and control groups. Applying Equation (11) to this hypothetical study, we see that, under the null hypothesis of equal probabilities, $\Pi_{{E_{i} }}$ and $\Pi_{{C_{i} }}$ are both equal to 0.35. Thus, due to ties, the nominal 0.50 value for $\Pi_{{E_{i} }}$ and $\Pi_{{C_{i} }}$ has been greatly reduced.

The EBT technique is indeed a “vote counting” method and such methods have been greatly disparaged by Rothman [16] among others as “methods to avoid”. However, unlike a simple Sign Test, the EBT method is based on a reasonable approach to the ties problem and combines the individual $P_{{E_{i} }}$ values by doing the equivalent of a formal convolution of the frequency distributions of the individual contributing studies.

### A non-parametric exact method for the estimation of effect size for dichotomous categorical meta-analysis

#### Basic estimation technique

**Fig. 3:**
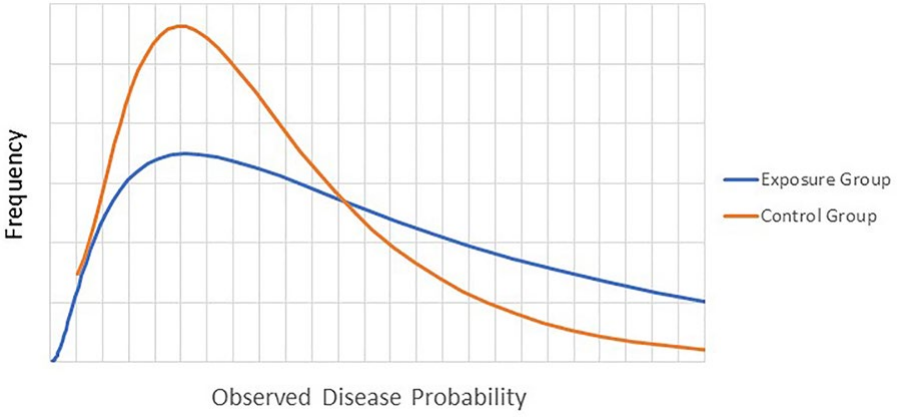
Demonstration of inappropriateness of simply directly comparing the $p_{E}$ and $p_{C}$ distributions to estimate Effect Size

A second exact technique was developed to estimate the effect size for dichotomous categorical meta-analysis. As a starting point, one might simply form the ratio of the average observed event probabilities, $p_{{E_{i} }}$ and $P_{{C_{i} }}$_*,*_ in the exposure and control groups respectively of each study and average these ratios across the *k* contributing studies. This simple approach, however, is highly biased. As shown in the underlying model that is described in Eqs. 1–4, the number of observed “successes” in the exposure and control arms of the *k* contributing studies each depend on an identical source of variation captured by $\varepsilon_{1i}$ in the model. The exposure group, however, contains an additional source of variation, captured by $\varepsilon_{2i}$ in the model. Figure 3 illustrates the problem of estimating the effect size by simply forming the ratio of $p_{E}$ to $p_{C}$.

Even for the relative risk of 1.0 depicted in the figure, the exposure distribution will have positive excursions that are not compensated for by equally robust negative excursions at least for small (rare) values of event probability.

The differential skew of the $p_{{E_{i} }}$ distribution relative to the $p_{{C_{i} }}$ distribution was used to address this issue. The additional skew in the exposed group due to the source of $\varepsilon_{2i}$ in Eq. 2 was estimated by taking the difference between the total exposure group skew and the expected skew from a pure binomial with the same observed event probability. The observed average $p_{E}$ across the *k* contributing studies was then reduced by a factor proportional to this difference in skew levels.

### Monte Carlo simulation of the ebt and dl techniques for statistical significance and effect size estimation

A series of Monte Carlo simulations was conducted to evaluate the EBT statistical significance test and the effect size estimation techniques and to compare them to the typically used DerSimonian–Laird Inverse Variance technique. The simulation was written and executed in the increasingly shared statistical language R [17]. The DerSimonian results were calculated using the “meta” package in R.

Five levels of relative risk (ratio of exposure group to control group event probability) of 1.0, 1.25, 1.5, 1.75, and 2.0 were crossed with three levels of disease background event probability (0.005, 0.01, and 0.05), and three levels of sample size (50, 100 and 200). Finally, the number of studies entering into each meta-analysis was chosen to be 5, 10, 20, or 40 studies. These choices allowed direct comparisons with the earlier work cited above ([12, 18]). In actuality, the background event probabilities were restricted to the small values that are typically encountered in epidemiological studies as discussed in Table 2.

In addition, the heterogeneity between the contributing studies, τ^2^ in Eq. 4, was evaluated at 0 (homogeneity), 0.4, and 0.8 to, again, allow comparisons to the earlier work. This last value of 0.8 represents a very large variance among the studies and was partially chosen to be able to compare the results with previous work. As an example, at τ^2^ = 0.8, a nominal exposure group event probability $p_{E}$ of 0.05 would vary from of 0.007 to 0.39 which is over a 35:1 ratio. Finally, the common variability in both the exposure and control groups represented by $\gamma^{2}$ in Eq. 1 was chosen to be 0.5 to again allow direct comparison with the earlier work.

The statistical significance and effect size were evaluated using both the EBT and DerSimonian techniques for each replication. All simulation runs were conducted with 10,000 replications. A value of 0.05 was used as the pre-specified level of Type I Error. The “Mid-P” technique advocated by Agresti [19] and others was used to determine the *p* values in a less conservative manner leading to more realistic power levels.

### Results from the Monte Carlo simulations: testing statistical significance

Figure shows the results of both the EBT and the DL methods. To simplify presentation, only scenarios in which the expected number of cases was greater than or equal to two were utilized. Table 4 shows the included scenarios.

**Table 4:** Scenarios included in the analysis of statistical significance

Background event probability	Sample size	Expected number of observed cases
0.01	200	2
0.05	50	2.5
0.05	100	5
0.05	200	10

**Fig. 4:**
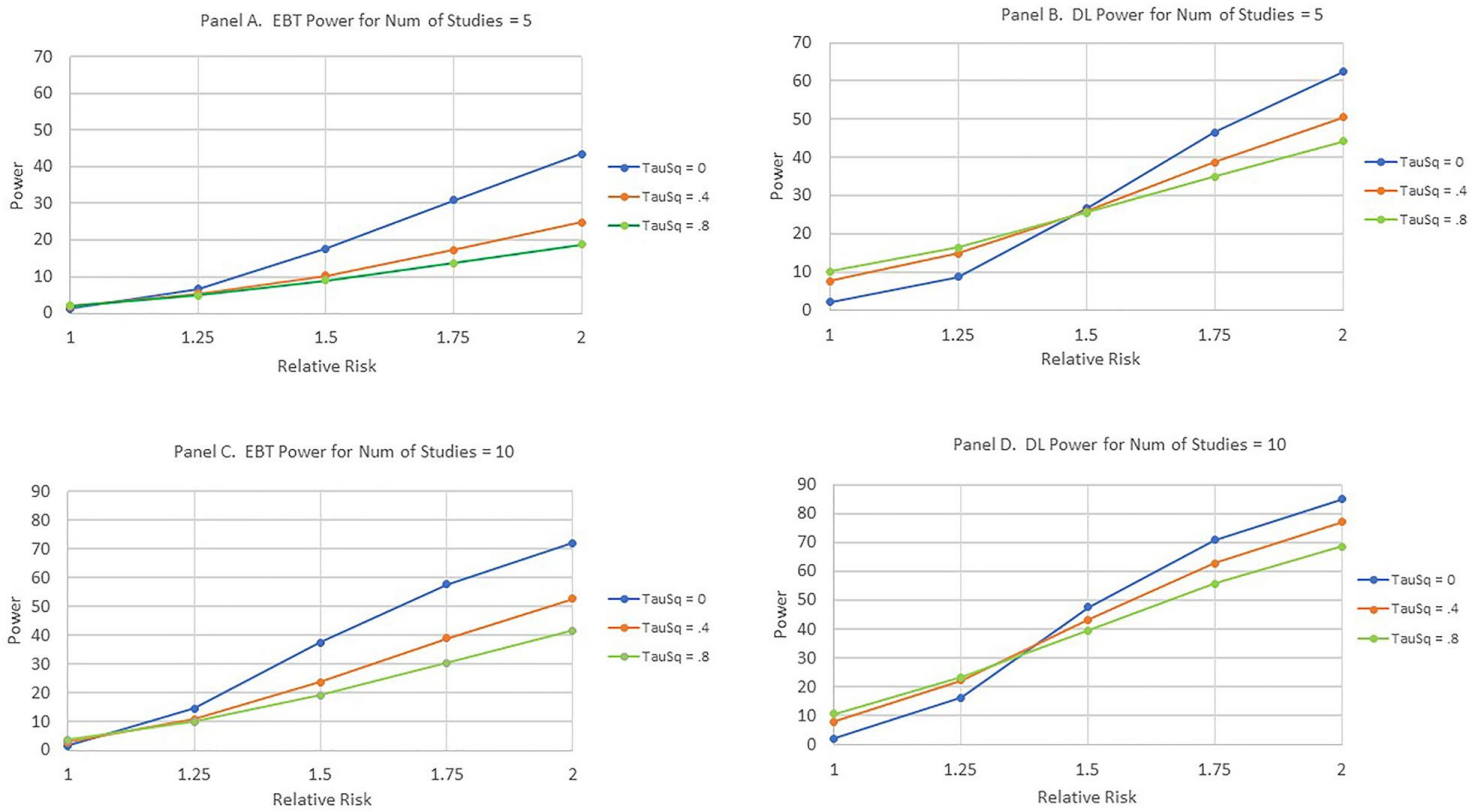

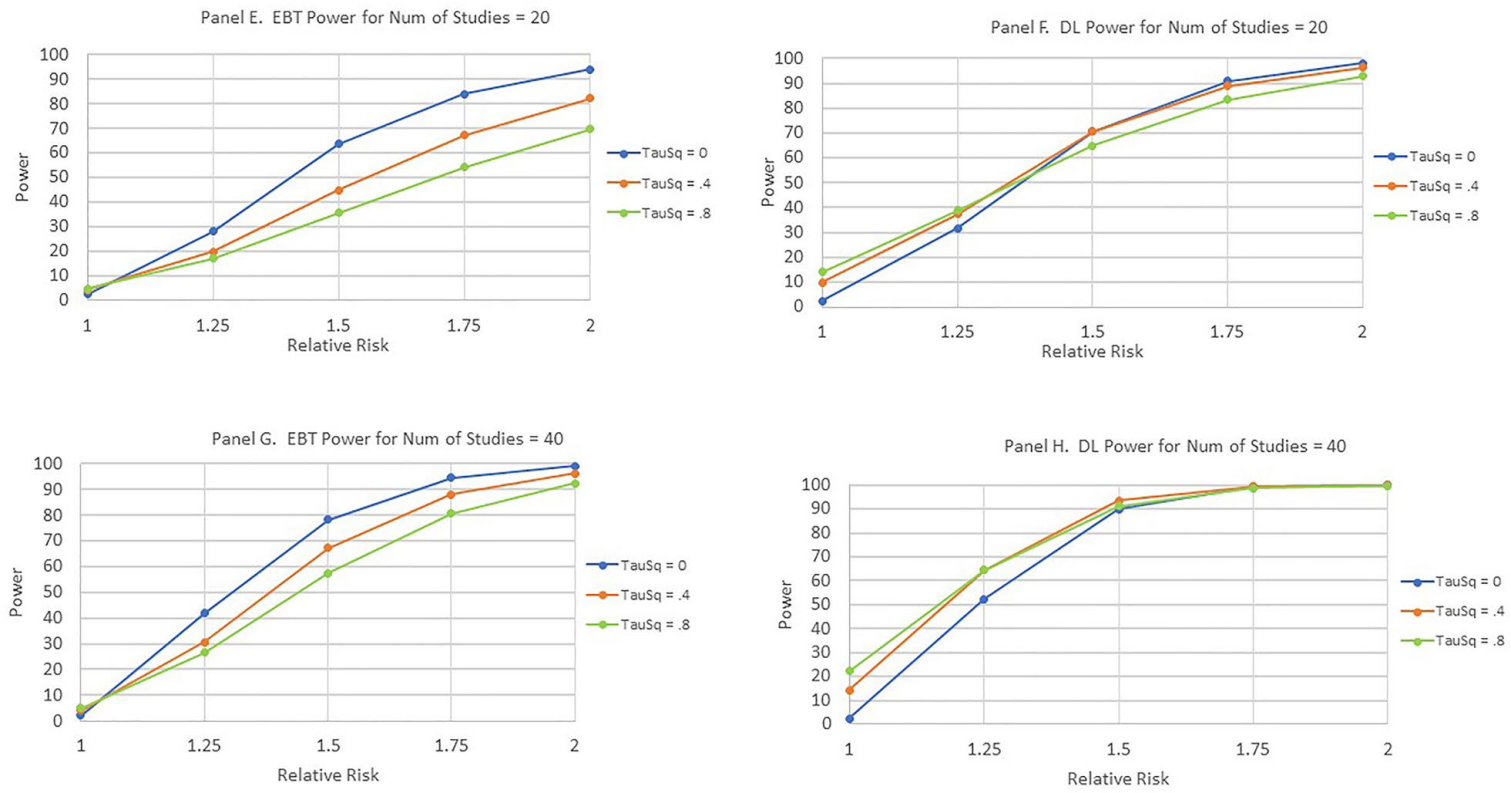
Power as a function of number of studies, relative risk, and heterogeneity. **A**, **C**, **E**, and **G** are for the EBT method and **B**, **D**, **F** and **H** are for the DL method

When the Relative Risk equals one, the power is the Type I error or, equivalently, the false alarm rate. The basic finding was that the EBT method maintained the prespecified level of Type I error for both the homogeneous and heterogeneous scenarios while the DL method had many violations of this level for heterogeneous scenarios. For the homogeneous scenario where τ^2^ = 0, both the EBT and the DL methods respect the prespecified Type I error level. However, for τ^2^ = 0.4 and for τ^2^ = 0.8, the DL method exhibits large violations of this level. As expected, as the number of contributing studies increases, the power for Relative Risk greater than one increases for both the EBT and DL methods. A separate analysis showed that the standard deviation of the power estimates in Fig. 4 was less than or equal to 0.42% (i.e., 0.0042).

In actuality, comparing the power between the EBT and DL techniques for Relative Risk ratios greater than 1.0 is not truly permissible due to the large number of violations of the pre-specified Type 1 Error for the DL technique.

**Fig. 5:**
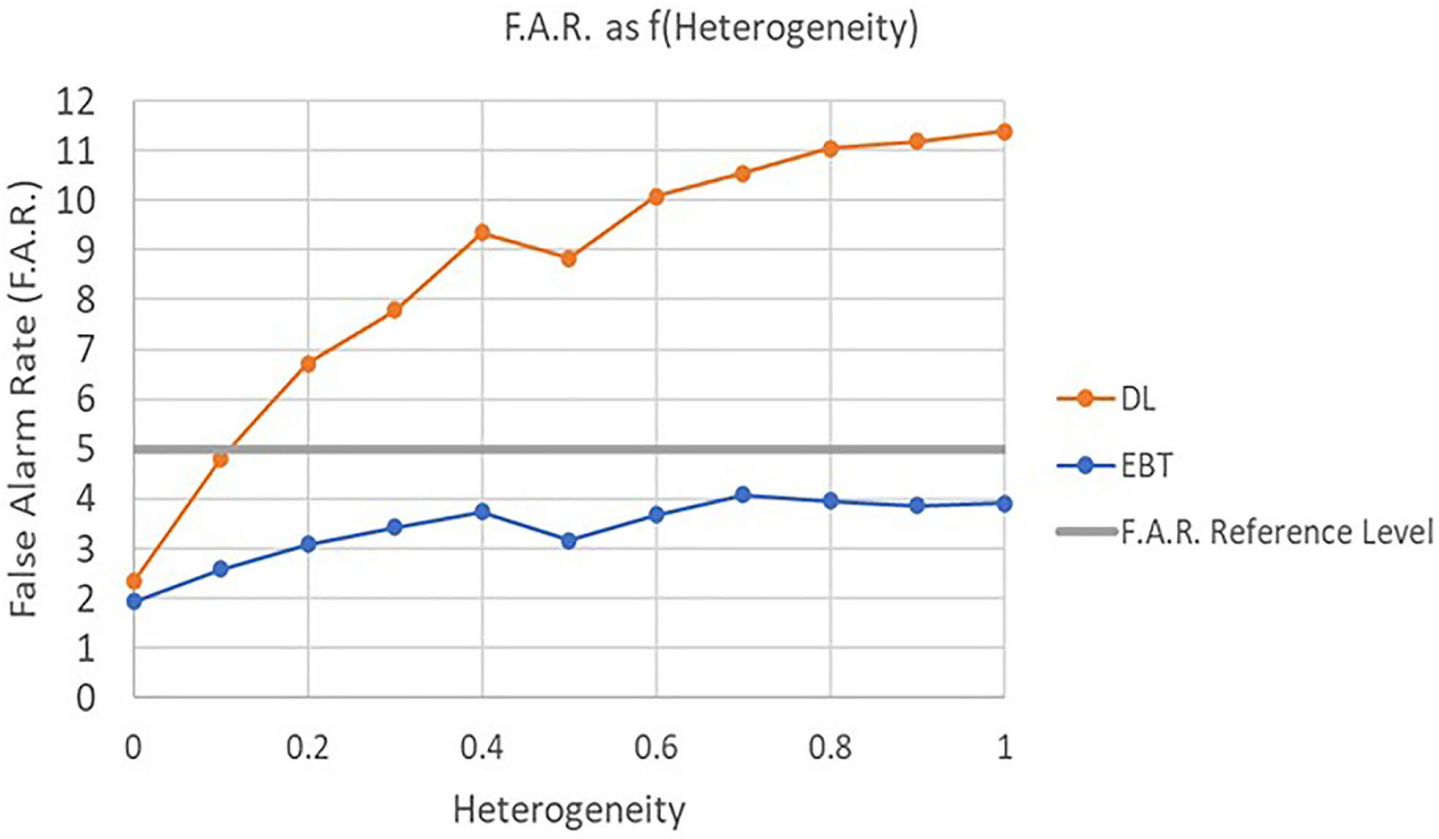
Type I error for EBT and DL methods as a function of heterogeneity

Figure 5 is a comparison of Type I Error (false alarm rate) for the EBT technique and the DL technique as a function of heterogeneity (τ^2^).

As can be clearly seen, the current EBT technique is relatively resistant to the effects of increasing heterogeneity over a very large heterogeneity range. The DL technique, however exhibits a monotonically increasing sensitivity to heterogeneity. A related aspect of any meta-analysis technique’s ability to perform well in the face of heterogeneity is its resistance to “contamination” from one or a small number of “rogue studies”. Since the EBT method does not directly allow such rogue studies to directly affect the test statistic, it should be much more resistant to these distortions.

The large costs of discreteness have been studied by Agresti [20] and others.

**Fig. 6:**
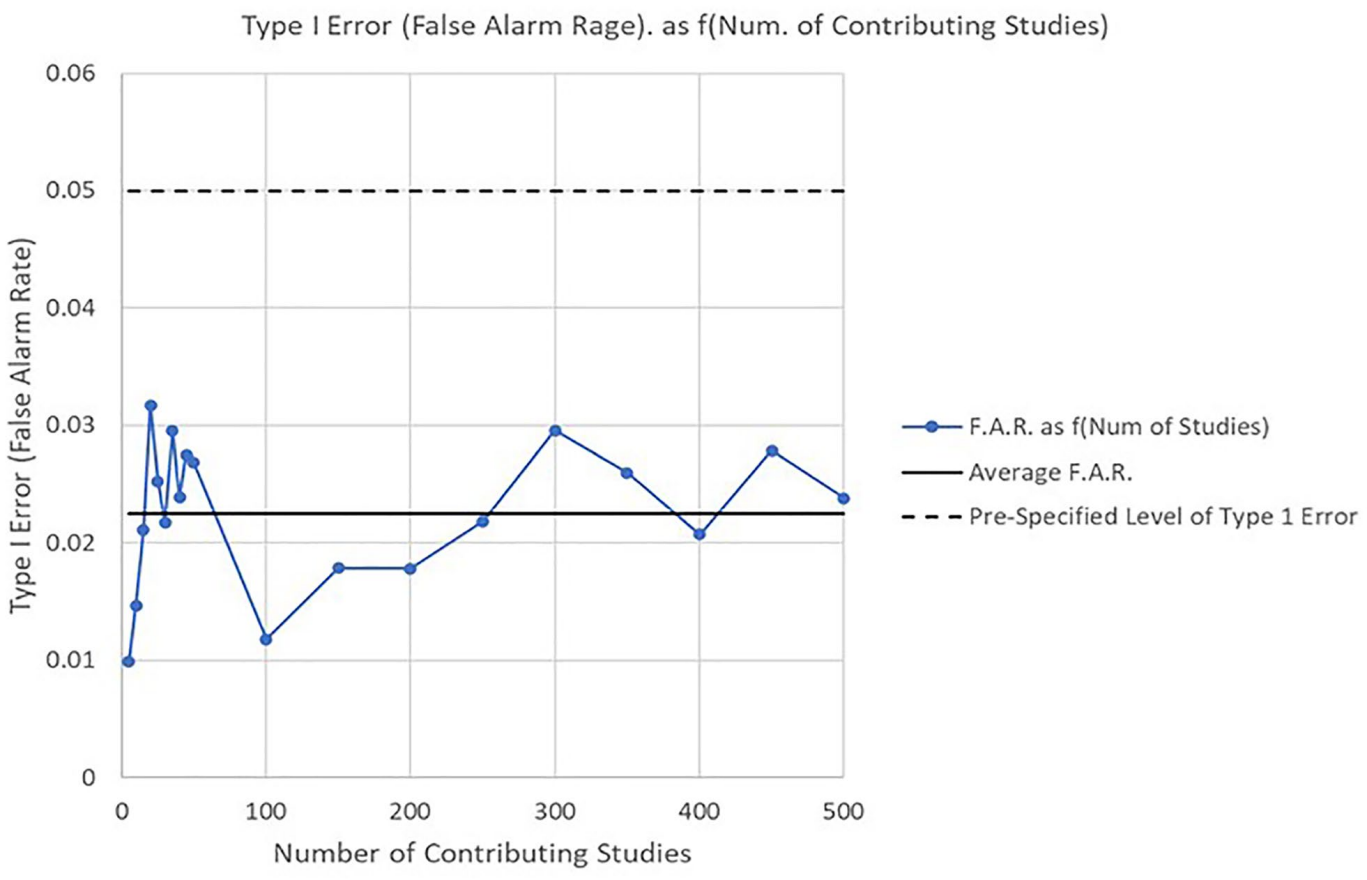
Interval overcoverage as a function of the number of contributing studies

A first cost of discreteness results when the number of contributing studies is small. The general issue of overcoverage is highlighted in Fig. 6.

The overcoverage is greatest for the smallest number of *k* contributing studies, and generally decreases as the number of contributing studies increases. As Fig. 6 demonstrates, even an unrealistic level of 500 contributing studies is still associated with a relatively large level of overcoverage. While such discreteness clearly reduces power, it could be argued that a statistically significant finding based on extremely sparse tables and a handful of studies requires stronger evidence. Unfortunately, the majority of meta-analyses consist of fewer than two or three studies as Kontopantelis et al*.* have shown in their extensive analysis of all meta-analyses in the Cochrane Library [21].

Additional Monte Carlo testing was done for unbalanced designs (unequal sample sizes in the exposure and control arms of the contributing studies) and meta-analyses with unequal sample sizes across contributing studies. Table 5 shows the sample sizes for the two groups for a typical unbalanced design in which the control group sample size is twice the exposure group sample size. The sum of the two sample sizes across both arms of the study was chosen to be 200 yielding an average sample size of 100 to allow comparison with the balanced designs of Fig. 4.

**Table 5:** Sample sizes for simulation of unbalanced designs

Group	Study #									
	1	2	3	4	5	6	7	8	9	10
Exposure	66	66	66	66	66	66	66	66	66	66
Control	134	134	134	134	134	134	134	134	134	134

Number of studies = 10

Table 6 below shows the results of the simulation for heterogeneity values τ^2^ = 0 and τ^2^ = 0.8, Event (“disease”) Probability of 0.05, Number of Studies = 10, and Sample Size (avg.) = 100 at the same five levels of Relative Risk used above. The simulation run consisted of 10,000 replications as in Fig. 4.

**Table 6:** Power (%) for the unbalanced design of Table 5 τ^2^ (heterogeneity) equal to 0 and 0.8; event probability = 0.05; Number of studies equal to 10; Sample size (per study arm) equal to 100

	Heterogeneity										
	0						0.8				
	Risk ratio										
Technique	1.0	1.25	1.5	1.75	2.0	1.0		1.25	1.5	1.75	2.0
EBT	2.1	14.4	43.3	71.0	88.4	4.2		11.0	21.6	35.0	46.9
DerSimonian and Laird	2.2	16.5	57.2	87.5	97.7	11.5		24.0	41.6	59.2	72.8

As the results in Table 6 show, when the heterogeneity was equal to 0.8, the Type I Error (Relative Risk = 1.0) remained below the specified value of five percent for the EBT technique but was far above this point for the DerSimonian.

Table 7 below shows the sample sizes for the exposure and control groups for each of the contributing studies for a design with unequal sample size across the contributing studies. This particular design was chosen as a relatively extreme case. As can be seen, the average sample size across the two groups was maintained at 100 to allow comparison of the simulation results with the equal sample size scenarios of Fig. 4.

**Table 7:** Sample sizes for simulation of unequal sample size designs

Group	Study #									
	1	2	3	4	5	6	7	8	9	10
Exposure	175	25	175	25	175	25	175	25	175	25
Control	175	25	175	25	175	25	175	25	175	25

Number of studies equal to 10

Table 8 below shows the results of the simulation for a heterogeneity values of τ^2^ = 0 and τ^2^ = 0.8, Event (“disease”) Probability of 0.05, and Sample Size (individual study arm average) = 100, at the same five levels of Relative Risk as used above. The simulation run consisted of 10,000 replications as in Fig. 4.

**Table 8:** Power (%) for the unbalanced design of Table 7 τ^2^ (heterogeneity) equal to 0 and to 0.8; Event probability = 0.05; Number of studies equal to 10; Sample size (avg. per individual study arm) equal to 100

	Heterogeneity									
	0					0.8				
	Risk ratio									
Technique	1.0	1.25	1.5	1.75	2.0	1.0	1.25	1.5	1.75	2.0
EBT	2.0	14.9	43.0	70.0	87.7	4.3	11.4	22.4	34.5	47.9
DerSimonian and Laird	2.4	16.6	56.6	87.2	97.8	11.4	25.1	41.6	58.2	73.5

Most importantly, at a heterogeneity level of 0.8, the EBT Technique was superior at protecting the pre-specified level of Type I Error relative to the DL technique.

A clear finding of the Monte Carlo simulations common to both meta-analysis techniques studies is the apparent fruitlessness of searching for small effect sizes. Both the EBT and DL techniques are very poor at reliably finding statistically significant results until the relative risk approaches 2.0. While this finding does not directly bear on the issues studied in this report, it does serve as a cautionary tale to those who continue to try to tease out very small effects especially from sparse data.

### Results from the Monte Carlo simulations: effect size estimation

**Fig. 7:**
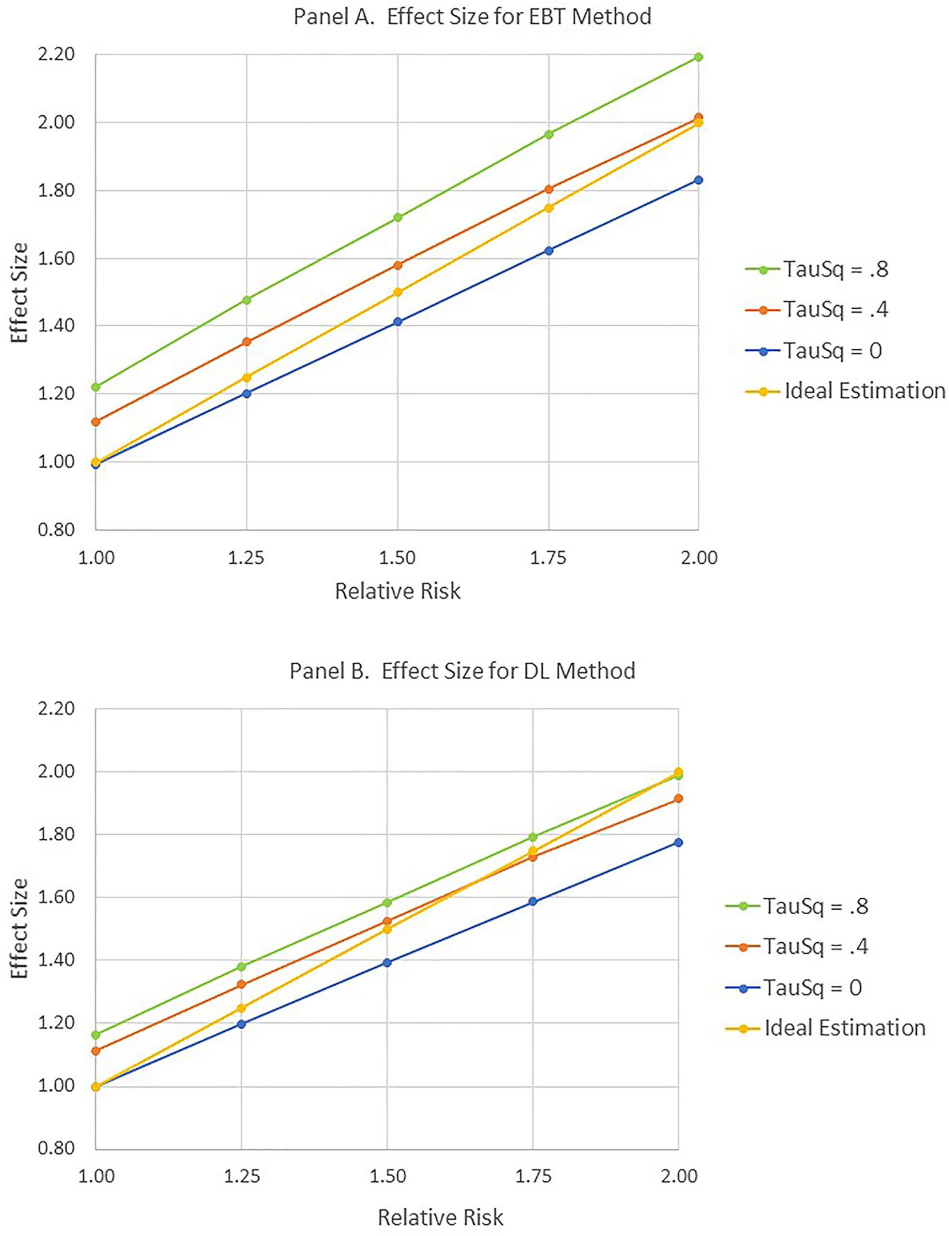
Effect size as function of relative risk and heterogeneity. **A** and **B** correspond to the EBT and DL methods respectively

**Fig. 8:**
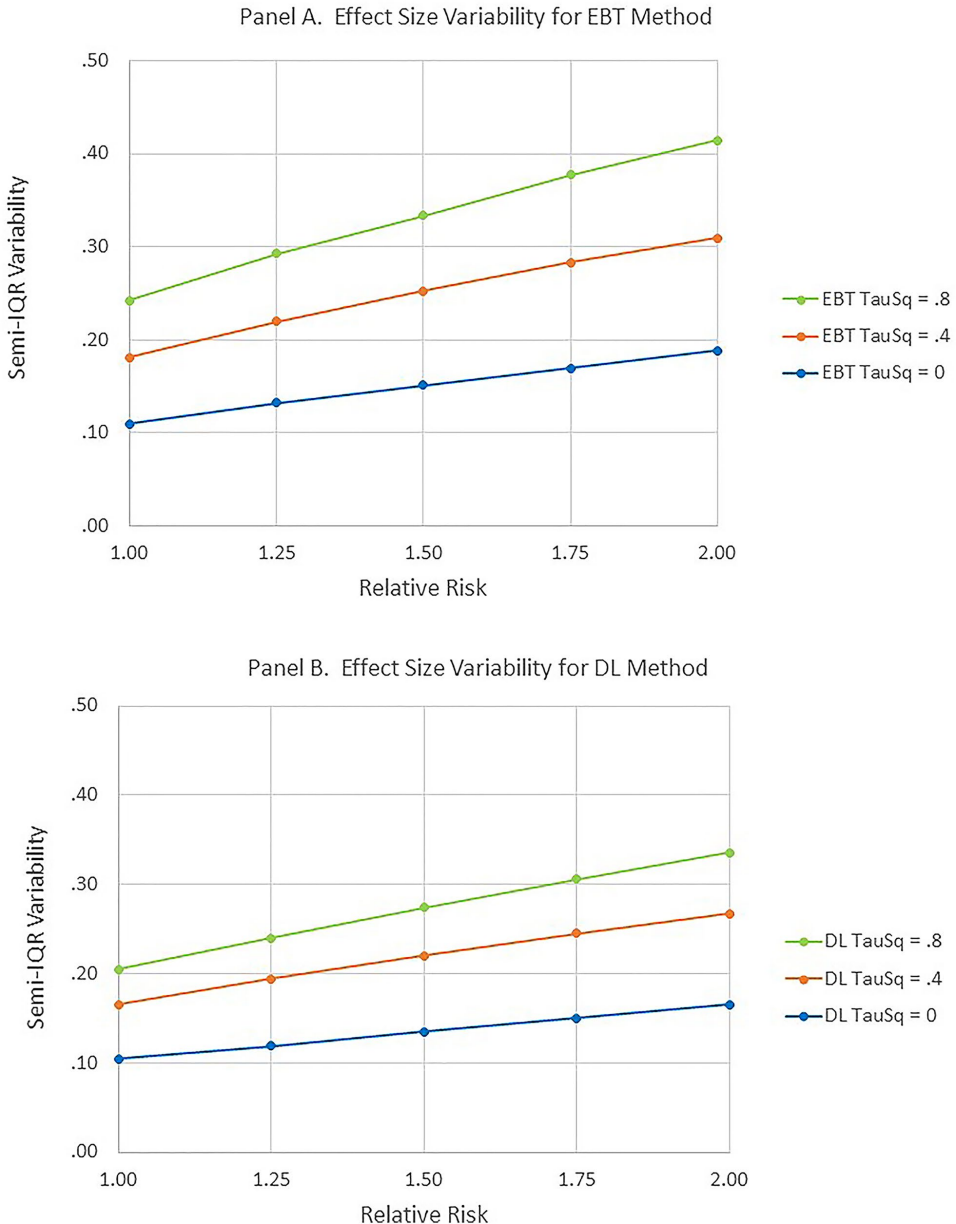
Semi-interquartile range as function of relative risk and heterogeneity. **A** and **B** correspond to the EBT and DL methods respectively

Figures 7 and 8 capture the basic findings for estimating the Effect Size.

Again, only simulation scenarios in which the expected number of observed cases was greater than or equal to two were utilized. Since the effect of the number of studies contributing to the meta-analysis was small for this effect size estimation, results were averaged across this variable. As shown in Fig. 7, both methods were reasonably successful at estimating the levels of relative risk. However, both methods generally underestimated the relative risk for τ^2^ = 0 and overestimated it for τ^2^ = 0.4 and τ^2^ = 0.8. Finally, as shown in Fig.  8, the interquartile range for the DL method was considerably smaller than for the EBT method.

## Conclusions and suggestions for the future

This research has developed an exact test for the meta-analysis of dichotomous, categorical data and a related method to estimate the size of the effect.

### The enhanced binomial technique (EBT) to assess statistical significance

The EBT technique was greatly superior to the DerSimonian technique in maintaining a pre-specified level of Type I Error. As shown, the DerSimonian technique demonstrated many large violations of this level when heterogeneity was present. Given the various biases towards finding statistical significance prevalent in epidemiology today, a strong focus on maintaining a pre-specified level of Type I Error would seem critical (see, e.g., [22]). The EBT approach is greatly superior at maintaining this pre-specified value of Type I Error in the face of even extreme heterogeneity.

### The enhanced binomial technique (EBT) to estimate effect size

A related but separate method was developed to estimate the effect size. This new technique was comparable to the often-used DL method although both methods demonstrated some accuracy issues. The DL method exhibited a somewhat smaller Semi-IQR variability. The fact that the EBT method was clearly superior in assessing statistical significance while the DL method demonstrated a smaller variability in estimating effect Size supports the possible utility of separating these two procedures as outlined at the beginning of this article. One possibility is to use the EBT method for statistical significance assessment and the DL method for effect size estimation.

While statistical programs providing exact solutions already exist such as Cytel’s StatXact, they are beyond the means of most practicing statisticians and epidemiologists. For example, Cytel Inc. currently lists a price of over $900 USD for their current version of StatXact 11 [23].

The techniques developed here are written in the almost universal statistical language of R and are freely available from the author. As such, it is hoped that other researchers would be able to extend and improve these initial versions.

As outlined in this report, the use of meta-analysis in epidemiology is increasing very rapidly and appears to be meeting an important need. Fortunately, inexpensive and readily available computer power has also vastly increased in the past forty years. For example, task speed as measured in Million Instructions per Second (“MIPS”) has increased from 0.64 for the IBM370 mainframe computer in 1972 to 238,000 for an Intel Pentium processor personal computer in 2014 [24]. By using the techniques developed here and the computer power available to all researchers today, the determination of statistical significance and the estimation of effect size can be readily accomplished without unnecessary error.

## Data Availability

Both software programs are freely available from the author.

## Footnotes

1 This number of combinations is simply C (24,12) = 2,704,156.

2 The probabilities for six or more ties decrease to extremely small values. However, in actuality, the EBT method calculates all possible ties in calculating $prob\left( {tie} \right)_{i}$.
